# Exploring the need for reconsideration of trial design in perioperative outcomes research: a narrative review

**DOI:** 10.1016/j.eclinm.2024.102510

**Published:** 2024-02-29

**Authors:** Henrik Kehlet, Dileep N. Lobo

**Affiliations:** a Section for Surgical Pathophysiology, Rigshospitalet, Copenhagen University, Copenhagen, Denmark; b Nottingham Digestive Diseases Centre, Division of Translational Medical Sciences, School of Medicine, University of Nottingham, Queen's Medical Centre, Nottingham, United Kingdom; c National Institute for Health Research Nottingham Biomedical Research Centre, Nottingham University Hospitals and University of Nottingham, Queen's Medical Centre, Nottingham, United Kingdom; d MRC Versus Arthritis Centre for Musculoskeletal Ageing Research, School of Life Sciences, University of Nottingham, Queen's Medical Centre, Nottingham, United Kingdom; e Division of Surgery, Perelman School of Medicine, University of Pennsylvania, Philadelphia, PA, USA

**Keywords:** Perioperative medicine, Enhanced recovery after surgery, Trial design, Interventions, Outcomes

## Abstract

“Enhanced recovery after surgery” is a multimodal effort to control perioperative pathophysiology and improve outcome. However, despite advances in perioperative care, postoperative complications and the need for hospitalisation and prolonged recovery continue to be challenging. This is further complicated by procedure-specific and patient-associated risk factors, given the increase in the number of elderly and frail patients with multiple comorbidities undergoing surgery. This paper is a critical assessment of current methodology for trials in perioperative medicine. We make a plea to reconsider the design of future interventional trials to improve surgical outcome, based upon studies of potentially effective interventions, but often without improvements in recovery. The complexity of perioperative pathophysiology necessitates a procedure- and patient-specific approach whenever outcome is assessed or interventions are planned. With improved understanding of perioperative pathophysiology, the way to improve outcomes looks promising, provided that knowledge and established enhanced recovery programmes are integrated in trial design.

**Funding:**

None.

**Research in context**

**Evidence before this study**

Postoperative complications and mortality are important considerations in healthcare given that more than 300 million operations are performed every year and there are more than 4 million deaths annually within 30 days of surgery. Consequently, research in perioperative medicine has proliferated in recent decades and has led to significant improvements in outcomes, especially those based on detailed knowledge and understanding of surgical pathophysiology, widespread introduction of minimal invasive surgery, and principles of enhanced recovery after surgery (ERAS). Nevertheless, postoperative complications, and the need for hospitalisation and prolonged recovery continue to be important challenges in relation to the many factors involved during the pre-, intra- and postoperative periods.

**Added value of this study**

Despite the demonstrated progress in surgical care, many studies on unimodal perioperative interventions have often failed to show appreciable clinically relevant improvement in outcomes. The duration of interventions to counteract the pathophysiological derangements should be considered in order to contribute to better outcomes. Consequently, in recent years we have witnessed several initiatives designed to improve and standardise outcome assessment and trial designs (large pragmatic randomised clinical trials, platform trials, detailed prospective cohort studies vs. pathophysiological mechanistic studies of postoperative organ dysfunction), all in order to accelerate the improvement of the knowledge base and limit the waste of research resources. This paper is a critical assessment of current methodology for trials in perioperative medicine. We make a plea to reconsider the design of future interventional studies to improve surgical outcome.

**Implications of all the available evidence**

The complexity of perioperative pathophysiology calls for a more procedure- and patient-specific approach whenever outcome is assessed or interventions are planned. There should be an emphasis on the prerequisite for an established, well-defined updated implemented multimodal ERAS programme for proposed trials. The duration of the intervention should be reconsidered compared with the time course of the pathophysiology of the specific outcomes in question in order to reduce the risk of later organ dysfunction.

## Introduction

**Fig. 1:**
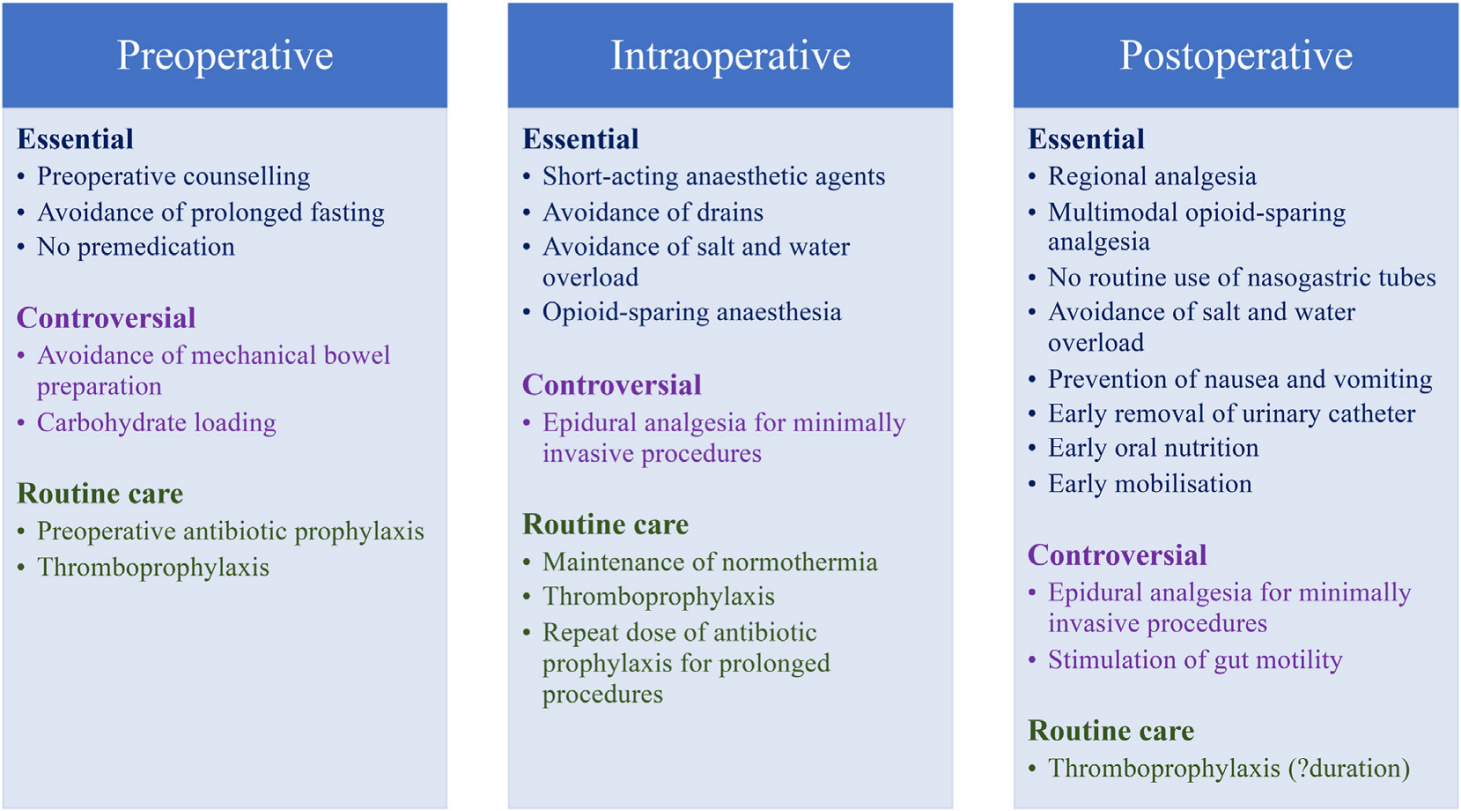
Elements of Enhanced Recovery After Surgery (ERAS) programme.

Postoperative complications and mortality are important considerations in healthcare given that more than 300 million operations are performed every year with more than 4 million deaths annually within 30 days of surgery.^1^ Postoperative mortality accounts for 7.7% of all deaths globally and surgery is the third most common cause of death after ischaemic heart disease and stroke.^1^ Consequently, research in perioperative medicine has proliferated in recent decades and has led to significant improvements in outcomes, especially those based on detailed knowledge and understanding of surgical pathophysiology, widespread introduction of minimally invasive surgery, and principles of enhanced recovery after surgery (ERAS) (Fig. 1).^2^ Nevertheless, postoperative complications, and the need for hospitalisation and prolonged recovery continue to be important challenges in relation to the many factors involved in perioperative care during the pre-, intra- and postoperative periods^3^ (Table 1).

**Table 1:** Perioperative factors to be considered for outcome improvement.

Preoperative	Intraoperative	Postoperative (in-hospital)	Post-discharge
Prehabilitation (should be combined with fully implemented ERAS programme. Prehabilitation may be more beneficial to patients who are frail and malnourished) Nutrition (should be combined with postoperative oral nutrition) Psychological (patients should be mentally prepared for surgery and the recovery process) Psychiatric (specific role of the psychiatric condition vs. pharmacotherapy) Preoperative anaemia optimisation	Minimally invasive surgery (study with well-defined ERAS programmes) Stress responses (inflammatory/neurohumoral modulation, regional analgesia, anti-inflammatory therapy (corticosteroids) Fluids (goal-directed fluid management for high-risk patients or high-risk procedures, choice of fluid, vasopressors, tissue perfusion Anaesthesia (short-acting agents) vs. postoperative pathophysiology Blood management (reduction of intraoperative bleeding (tranexamic acid/minimally invasive surgery), optimise patient-specific transfusion thresholds)	Cognitive function (role of pain, sleep, inflammation, opioids, other drugs) Orthostatic intolerance (mechanisms, prevention) Thromboembolic prophylaxis Ileus (objective assessment of resolution in specific patient populations with well-defined ERAS programmes) Sleep (pain control, noise reduction, inflammation reduction) Why in hospital? (detailed analysis of factors responsible)	Objective activity assessment Quality of life Quality of recovery (return to normal function) “Days alive out of hospital” (surgery-related vs. disease or other reasons) Complete follow-up (separate “medical” vs. “surgical” complications) Mortality (surgery-related vs. other reasons) Patient-reported outcome measures (PROMs) Extended thromboembolic prophylaxis (is it necessary?) Effect of postoperative anaemia on functional recovery Pain management (prevention of chronic post-surgical pain and persistent postoperative opioid use)

The problem is further complicated by procedure-specific issues and patient-associated risk factors, given the increase in the number of older patients, those with frailty, and those with multiple comorbidities undergoing surgery.^4^^,^^5^ Nevertheless, despite the demonstrated progress in surgical care, many studies on unimodal perioperative interventions have often failed to show appreciable clinically relevant improvements in outcomes.^6^ Consequently, in recent years we have witnessed several initiatives designed to improve and standardise outcome assessment^7^^,^^8^ and trial design [large pragmatic randomised clinical trials (RCTs), platform trials, detailed prospective cohort studies and pathophysiological mechanistic studies on postoperative organ dysfunction], all in order to accelerate a knowledge base for the development of procedure-specific outcome improvement programmes.

This paper is a critical assessment of current methodology for recent trials in perioperative medicine. We make a plea to reconsider the design of future interventional studies to improve surgical outcome. We emphasise the prerequisite for an established, well-described and implemented multimodal ERAS programme for proposed trials. In this context, the potential of a short-term intervention should be reconsidered based upon the pathophysiology of the specific outcomes in question compared with the duration of the intervention and potential effects on the stress responses to surgical injury and subsequent risk of organ dysfunction.

The main argument for a need for improved strategy for outcome research design is based upon the realisation that the combined injury-induced inflammatory, immunological, endocrine and metabolic responses are the key factors leading to the risk of organ dysfunction, complications and impaired recovery,^9^ but with an important plethora of additional contributing factors^3^ listed in Table 1. Consequently, the concept of ERAS represents a multimodal intervention that has been proven to be effective in reducing the risk of “medical” complications by 30–50%,^2^^,^^3^ and with a higher compliance with the ERAS components leading to better outcomes.

Thus, our arguments for a reconsideration of trial design are based upon the many interventional studies on potentially effective interventions, but often without significant improvements in recovery as illustrated below for many pathophysiological mechanisms of morbidity and recovery or interventional techniques as well as a lack of details on an ERAS programme and its implementation.

However, although a “full” ERAS implementation is warranted, the reality is that there are several international versions of ERAS guidelines which often include many variable components that are not all based on existing evidence^10^ which make definite recommendations for ERAS programmes in the future difficult. Hence, future trials should specify the ERAS elements employed and the results in the study group should be compared with the best available data from other published procedure-specific programmes in order to reduce noise and improve interpretation. Future studies should also avoid a general “compliance implementation percentage”, but instead divide it into the different pre-, intra- and postoperative elements which have a variable clinical relevance.

## Search strategy and selection criteria

We searched the PubMed database with the terms “perioperative care”, “enhanced recovery after surgery”, and “fast-track surgery”, in combination with one or more of the terms “clinical trials”, “systematic reviews”, “meta-analyses”, “reviews”, “outcomes”, “pathophysiology”, “compliance”, and “acceptability” for articles published from January 1, 1995 to January 31, 2024. We identified articles on adult surgical inpatient populations and selected the most relevant clinical trials, systematic reviews, and high-quality narrative reviews mainly published from 2019 to 2024. We also manually searched reference lists of identified articles to retrieve additional studies and included a few older articles if relevant.

## Preoperative considerations

### Prehabilitation

The rational approach with *prehabilitation* in patients with limited functional reserve capacity has mostly been disappointing, probably because the achieved improvement in preoperative function was not combined with a fully implemented ERAS programme resulting in a traditional loss of postoperative function and lack of the benefits achieved preoperatively.^11^^,^^12^ Additionally, interventions for prehabilitation have varied between unimodal and multimodal ones, duration and intensity of interventions, and patient populations. There is also a need for reconciliation between the optimal duration of the intervention and the delay in treatment caused by the intervention as it has been shown that for every 4-week delay in the treatment of some visceral cancers, there is a decrease in long-term survival.^13^ However, promising data from studies within an implemented ERAS programme suggest a benefit from prehabilitation^14^ calling for similar studies in high-risk patients across procedures. Prehabilitation also seems to have a better effect on outcome when it is applied to older adults and those with frailty.^15^

### Anaemia

*Preoperative anaemia* is a well-documented surgical risk factor^16^^,^^17^ and, therefore, needs to be managed. However, until now most studies have not demonstrated improvements in outcome,^18^^,^^19^ again probably associated with lack of a combined ERAS approach,^20^ especially in patients who are at high-risk.^21^ In this context, future studies should also consider a potential revision of traditional transfusion guidelines for patients with anaemia who are at high risk and not manageable with medicines alone.^17^

## Intraoperative factors

### Inflammatory and immunological responses

In recent years, it has become increasingly clear that even with well-defined standard surgical procedures, the magnitude of the response to surgical injury is patient-specific and involves a plethora of inflammatory and immunological responses which have been shown to be related to postoperative recovery measures such as fatigue, pain and hip function.^9^ These changes may last for days, making it apparent that certain future interventions should have a duration of several days or more. Interestingly, similar studies have shown the ability to identify patients who are high inflammatory responders or at high risk preoperatively based on a detailed analysis of white cell function.^22^ Interestingly, such preoperative analyses have been shown, in preliminary studies, to predict the risk of surgical site infection.^23^ In future large-scale studies, simple and inexpensive C-reactive protein assessment may be sufficient, although more detailed components may be required in smaller hypothesis-generating studies. More recently, attention has been drawn to the role of post-injury autonomic dysfunction in promoting organ dysfunction.^24^ Neuromodulation (parasympathetic) may potentially reverse this dysfunction and should be studied further.^24^

Currently, three techniques are available to modify surgical stress responses where afferent neural blockade techniques predominantly modify the hormonal responses (and pain), but less so the inflammatory responses where minimally invasive surgical techniques and glucocorticoids are more effective.^3^ However, undesirable stress responses may still occur in certain patients, with those thought to have a high risk being the most susceptible. Presently, these proinflammatory immunological responses may only be attenuated by using high doses of glucocorticoids preoperatively as shown in many studies, including minimally invasive procedures like endovascular aortic repair.^25^ These responses can be significantly reduced by administering high-dose steroids to blunt the systemic inflammatory response syndrome that leads to delayed recovery.^25^ Many other studies have highlighted the potential benefits of preoperative steroids on aspects of recovery. Safety has been emphasised in these studies, but a procedure-specific approach is needed with more dose-finding studies, and identification of patients who are “high-inflammatory” responders.^26^^,^^27^

### Analgesia

The concept of *pre-emptive analgesia* is based up on the hypothesis that a preinjury blockade of the nociceptive input may reduce postoperative adverse organ responses including acute and persistent postoperative pain. Although attractive, unfortunately the clinical studies have been rather dispappointing,^28^ probably explained by the short-duration of the pre-emptive techniques compared with the duration of the post-injury inflammatory and immunological responses important for recovery.^9^ In contrast, “preventive analgesia” should be provided, i.e., effective analgesia when the patient wakes up, but continued until sufficient pain relief has been achieved and with the primary aim being to reduce acute postoperative pain. Thus, preventive analgesia with a focus on pain relief remains a prerequisite for improved outcome and should be continued and documented until reasonable functional recovery has been achieved.^3^

The concept of *opioid-free anaesthesia* is, in theory, rational because opioid-related side effects delay recovery. However, three recent RCTs on opioid-free anaesthesia have not shown any benefit when compared with standard protocols.^29^, ^30^, ^31^ Also, an editorial^32^ accompanying one of the studies^29^ concluded that although opioid-free anaesthesia was feasible, it was neither logical nor beneficial to patients, suggesting that opioid-sparing anaesthesia rather than opioid-free anaesthesia may be the way forward. However, such future studies should still include a focus on an effective ERAS programme.

### Intravenous fluids and haemodynamic monitoring

During the last two decades, numerous studies on perioperative fluid therapy have shown that too much or too little fluid, even during uncomplicated surgery, has detrimental effects on recovery^33^, ^34^, ^35^ and this has been confirmed in a large pragmatic RCT.^36^ However, currently the main discussion is about the principles of effective haemodynamic monitoring which remains debatable,^37^ and more importantly about the controversial topic of goal-directed haemodynamic therapy where we probably need to be more precise.^38^ The concept of goal-directed haemodynamic management was initially based on early intraoperative stroke volume optimisation and where some of the many RCTs have suggested some improvements in recovery and postoperative morbidity.^39^ However, all these studies suffer from methodological problems; first of all including disperse types of patients and surgical procedures and a surprising lack of information about perioperative care and adjustment to current evidence on the results of ERAS, and the neglect of the importance of perioperative fluid management and/or goal-directed haemodynamic therapy when the patient has returned to the ward. Most studies do not provide details on postoperative intravenous fluid therapy and the majority used 6% hydroxyethyl starch for bolus therapy, the use of which is now restricted because of potential renal adverse events. Moreover, relatively recent meta-analyses have shown that although goal-directed fluid therapy improves outcome in patients managed with traditional care pathways, this benefit is not apparent in those managed with ERAS pathways.^40^^,^^41^ A number of different technologies have been used for haemodynamic monitoring and it is still not certain which is the best.^42^ There are also substantial differences in target variables for haemodynamic therapy, target values, and triggered interventions, and this variability could have different effects on outcome.^38^ Thus, future studies on haemodynamic therapy are required with an optimised design in specific well-defined high-risk patients and in well-defined procedures which on their own have different fluid dynamics. Participants in these studies should be included in a defined and monitored implemented ERAS programme for the procedure in question in order to provide better answers to this important problem.

### Delirium and cognitive dysfunction

*Postoperative delirium and cognitive dysfunction* are well known, relevant and accepted outcome measures.^43^ Despite being extensively investigated, the many single modality interventions for this complex problem remain debatable.^43^ Again, most of the available studies suffer from the methodological problems of unimodal interventions for a multifactorial problem including neuroinflammation, pain, sleep disturbances and use of opioids. However, the vast number of RCTs have not corrected for these pathophysiological factors. In contrast, a few surgery-specific (orthopaedic and colonic surgery) cohort studies with inclusion of an effectively implemented ERAS programme and early return to home suggest that these problems are smaller than what has been reported in previous studies. Therefore, the road forward is to establish ERAS programmes before studying single or multiple interventions to reduce delirium and late cognitive dysfunction in certain high-risk patients or procedures.^44^

### Minimally invasive surgery

*Minimally invasive surgery* is obviously a rational choice because of the lesser magnitude of trauma with reduced pain, and inflammatory and immunological responses resulting in an expected accelerated recovery. Again, results from observational studies and a few RCTs suggest outcome benefits of minimally invasive surgery irrespective of the technique used (laparoscopic, thoracoscopic, robotic, etc.). Nevertheless, from a strict scientific point of view most of these observations suffer from an influence of an unblinded setup which may introduce major bias from the surgeons, nurses, and other healthcare professionals, influencing patient recovery, which of course is no criticism if the outcome is improved. However, as shown in an initial study comparing mini with laparoscopic cholecystectomy,^45^ no difference in outcome was demonstrated because the outcomes considered were not analysed with regard to the surgical technique vs. the whole traditional perioperative care setup. Only a few double-blind studies securing patient and surgical round blinding because of a dressing over the abdomen after laparoscopic vs. open colonic resection or robotic cystectomy^46^^,^^47^ are available together with an established effective ERAS programme, with length of stay about of 2 and 5–6 days respectively. However, early recovery could not be demonstrated to be improved by the minimally invasive approach before removing the surgical dressing. Nevertheless, these observations are not arguing against minimally invasive surgery, but for a reconsideration of design of future studies with a combination of improved prolonged multimodal opioid-sparing analgesia together with further pharmacological interventions on the inflammatory responses to promote recovery. Furthermore, minimally invasive surgery may have benefits other than early recovery by reducing surgical site wound infections, hernia formation, etc.

## Postoperative factors

Optimised *pain management* is an obvious prerequisite for further improvement in outcome, but despite a large number of studies, including RCTs, we still have a major clinical problem that requires further investigation and improvement. This implies a reconsideration of pain assessment where, unfortunately, only a few RCTs include pain assessment with well-defined, procedure-relevant function. Also, in the many RCTs it is difficult to explain the value of individual pain-relieving techniques, since the concomitant use of other evidence-based analgesics has most often not been standardised or implemented.^48^ The way forward is to focus on procedure- and patient-specific approaches, since pain and its influence on recovery is both procedure- and patient-dependent.^49^ Patients who are pain catastrophisers, preoperative opioid users and high inflammatory responders may be more prone to an exaggerated postoperative pain response.^50^ Although these considerations are important, many studies on postoperative pain management have neglected these aspects and, consequently, have limited the possibilities for improved interpretation and progress. In addition, unimodal pain assessment techniques such as the visual analogue scale should probably be replaced with functional pain scores in order to rationalise the delivery of analgesics.^51^

## Outcome assessment

Despite the many approaches for a definition of perioperative medicine endpoints of morbidity and organ failure,^7^ there is a need for further standardisation and use of agreeable outcomes, which often have been different in anaesthesia vs. surgery directed studies.^8^ Previously, in most studies length of postoperative hospital stay has been an outcome which although relevant, is unfortunately rather nonspecific and depends on many factors other than the physiological recovery process. Nevertheless, future outcome studies on hospital stay are important provided that they are performed with detailed analysis of the reasons for patients not being discharged.^52^ Also, despite the rational approach with patient-reported outcome measures (PROMs), more trials are needed to compare them with objective assessment of recovery where the latter may be less positive than PROMs, but where the combined outcome assessment may help to understand reasons for impaired post-discharge activity (i.e., pain, fatigue, etc.).^53^^,^^54^ Furthermore, there should be greater focus on post-discharge functional recovery, rather than hospital stay alone. In this context, assessment of overall healthcare burden like “days alive and out of hospital” (DAOH),^55^ including readmissions are relevant. Finally, future trials should desist from reporting overall postoperative mortality and instead differentiate between potentially modifiable surgery-related mortality vs. disease-related mortality (i.e., cancer, etc.) vs. others (i.e., road traffic accidents, etc.).^56^ Composite end points, such as combining morbidity with mortality may help reduce sample sizes and make trials more manageable and deliverable.

Because of the plethora of different outcomes, there have recently been efforts to reach consensus definitions for standardised endpoints in perioperative medicine,^7^ but with different consensus from other stakeholders with a dominant focus on specific complications and consequences.^8^ Hopefully, such viewpoints may soon lead to a common consensus on how to define postoperative outcome endpoints on a procedure- and patient-specific basis.

### Unimodal interventions

Perioperative and surgical care is complex, and interventions have to be provided across the continuum of the pre-, intra- and postoperative phases. With the level of complexity involved, gains produced by unimodal interventions may be marginal and not result, on their own, in a significant improvement in postoperative outcomes. However, when several interventions with marginal gains are combined, the net improvement is greater than the arithmetic sum of the benefits of each individual intervention. Similarly, inclusion of several interventions with potentially deleterious effects or exclusion of potentially beneficial interventions can lead to a cumulative deterioration in outcomes.^57^

### Interventions and participants

Although large scale, multicentre RCTs are the current gold standard for effecting change, it is important that the magnitude of the intervention and the participant population should be appropriate to answer the question being asked. This has been evident in some trials, especially those on prehabilitation, where there is a large heterogeneity in the population of patients studied, the type and duration of intervention and the recording of compliance with the intervention. An ineffective intervention offered to participants who do not need it is very unlikely to produce appreciable benefit. Moreover, interventions that require a long duration to produce a positive effect (preoperative anaemia correction, exercise, etc.) may delay surgery for cancer and this may negate the long-term benefits and even cause harm.^13^ Inadequate or inappropriate doses of medicines or volumes of intravenous fluids may also show lack of benefit. Aspects of care other than the intervention should also be optimal, as interventions are unlikely to show benefit if the overall standard of care is suboptimal. In addition, in many trials where no difference has been shown in primary outcome measures, recommendations for change in practice have been made on the basis of secondary outcome measures and sub-group analyses when, in fact, the latter two should have directed design of future trials. Emphasis should be made on clinically relevant improvements in outcomes rather than merely statistically significant differences.

Previously, many outcome trials have been designed as large, pragmatic RCTs which are costly and have taken a long duration to deliver, but due to the many confounding factors and not including well-defined and implemented ERAS programmes,^58^ these have often been “negative” meaning that the effect of the unimodal intervention studied was not statistically significant. However, large pragmatic RCTs represent real world data which obviously are of value to verify the present quality of treatment, but less valuable to make progress. As an example, if the power analysis in such a pragmatic RCT is based on an expected 15% effect of the intervention, but when ERAS is instituted a >30% outcome effect may be expected.^2^ Consequently, if ERAS is not instituted it leads to an inherent risk of noise and, again, a risk of a negative trial. On the other hand, interventional studies may require a larger sample size when ERAS is implemented in both arms.

In order to avoid these problems, a newer design approach has been the “platform trial”,^59^ which may be more surgery-specific, to simultaneously compare several interventions in a shorter time and, therefore, accelerate accumulation of knowledge and change in clinical practice. The platform trial design includes sharing of a single control group but including several treatment groups, thereby, decreasing the overall required sample size and with an option to stop or add new treatment options, thereby providing a potential to accelerate knowledge acquisition.^59^ However, such a design should still include an ERAS approach which has been demonstrated to be beneficial across procedures, although still with challenges for improvement.^2^^,^^3^

### Acceptability of interventions to patients

There is an increasing requirement to involve patients with lived experience of the condition to be involved with clinical studies. This is a positive move as it helps provide information about acceptability of the intervention to the patient and also helps determine which patient-relevant outcomes should be studied.

### Compliance with interventions

Although compliance with the intervention should be recorded, analysis of results of trials should be based on an intention to treat analysis. This is important as in real world clinical situations not all patients are compliant, and efficacy of the intervention should be based on the lowest common denominator. Pre-specified subgroup analyses may be performed on participants with high and low compliance.

## Statistical considerations

Even though the same treatment can have varying impacts in different participants and populations, RCTs and observational studies that compare effectiveness of interventions usually report an average treatment effect that is a summary of individual treatment effects. Differences in characteristics of patients can lead to heterogeneous responses to interventions. Variations that are often undesirable in studies can be reduced by excluding participants with characteristics thought to cause variations in responses,^60^ but this intentional restriction in heterogeneity of patient populations within RCTs limits generalisability. Determining whether a treatment works for people in a target population that differs from the study population requires additional information and methods.^60^ However, at the same time, selecting participants who are not in need of an intervention can reduce the effect size of the intervention (e.g., offering intravenous iron to participants with a normal haemoglobin and ferritin, and offering prehabilitation to athletes).

This problem may be overcome by heterogeneity of treatment effect analyses to estimate treatment effects in clinically relevant subgroups (subgroup analysis) and to predict whether an individual might benefit from a treatment (predictive learning).^60^ Although pre-determined subgroup analysis may help overcome this, it should be remembered that creation of subgroups lowers the power to detect differences in subgroup effects.

Heterogeneity may be more pronounced in pragmatic trials, with between-patient variability being the main source. Hence, the appropriate design approach for each domain should aim at matching the overall intention while optimising the balance between desirable and undesirable heterogeneity.^61^

## Conclusion

In summary, the complexity of perioperative pathophysiology calls for a more procedure- and patient-specific approach whenever outcome is assessed, or interventions are planned. In very large pragmatic RCTs with many confounding factors, the duration of interventions to counteract the pathophysiological derangements should be considered in order to contribute to better design and more reliable outcomes. Hopefully, such an approach will also lead to better understanding of postoperative outcome pathophysiology by avoiding transference of data and knowledge from one procedure to another and furthermore include a better patient-specific approach regarding not only conventional specific risk factors, but also inflammatory and immunological characteristics, as well as specific characteristics that determine response to pain (high vs. low pain responders). Thanks to the improved understanding of the many aspects of perioperative pathophysiology, the way forward to improving outcomes looks promising, provided that knowledge is integrated in future outcome trial design (Table 2).

**Table 2:** Factors to be considered in future surgical outcome trial design.

• No trials should be undertaken without a well-designed procedure-specific updated enhanced recovery after surgery (ERAS) programme combined with compliance reporting of the specific pre-, intra-, and postoperative ERAS elements
• The duration of intervention should be compared with expected postoperative pathophysiological changes and risk of organ dysfunction
• Procedure-specific approach and patient-specific approach (e.g., “high” vs. “low” pain and “inflammatory responders”) should be implemented
• Argue for the pathophysiological role of a single intervention within a multimodal intervention (ERAS)
• Focus on post-discharge recovery, complete follow-up, including quality of life and quality of recovery
• Initial pilot observations based on perioperative pathophysiology and organ (dys)function are necessary before “big” trials

## Outstanding questions

Due to the complexity of perioperative pathophysiology and risk of organ dysfunction and complications, future trials should include a procedure- and patient-specific approach combined with a well-defined and implemented enhanced recovery (ERAS) programme. Detailed pilot observations are recommended before designing “big” trials.

## Contributors

Both authors contributed equally to this article, had access to the cited literature and are responsible for the decision to submit the manuscript.

## Declaration of interests

HK has no conflicts of interest to declare. DNL has received an unrestricted educational grant from B Braun and speaker's honoraria from Nestlé, Abbott and Corza for unrelated work. DNL is also the Scientific Chair of the ERAS® Society.

## References

[1] Nepogodiev D., Martin J., Biccard B., Makupe A., Bhangu A., National Institute for Health Research Global Health Research Unit on Global Surgery (2019). Global burden of postoperative death. Lancet.

[2] Ljungqvist O., de Boer H.D., Balfour A. (2021). Opportunities and challenges for the next phase of enhanced recovery after surgery: a review. JAMA Surg.

[3] Kehlet H. (2020). Enhanced postoperative recovery: good from afar, but far from good?. Anaesthesia.

[4] (2018). Administration for community living and administration on aging.

[5] Fowler A.J., Abbott T.E.F., Prowle J., Pearse R.M. (2019). Age of patients undergoing surgery. Br J Surg.

[6] Sessler D.I. (2020). Negative trials, and what to do with them?: first, stop calling them "negative". Anesthesiology.

[7] Jackson A.I.R., Boney O., Pearse R.M. (2023). Systematic reviews and consensus definitions for the standardised endpoints in perioperative medicine (StEP) initiative: mortality, morbidity, and organ failure. Br J Anaesth.

[8] Abbassi F., Walbert C., Kehlet H., Grocott M.P.W., Puhan M.A., Clavien P.A. (2023). Perioperative outcome assessment from the perspectives of different stakeholders: need for reconsideration?. Br J Anaesth.

[9] Gaudilliere B., Fragiadakis G.K., Bruggner R.V. (2014). Clinical recovery from surgery correlates with single-cell immune signatures. Sci Transl Med.

[10] Kehlet H., Memtsoudis S.G. (2020). ERAS guidelines for hip and knee replacement - need for reanalysis of evidence and recommendations?. Acta Orthop.

[11] Lobo D.N., Skořepa P., Gomez D., Greenhaff P.L. (2023). Prehabilitation: high-quality evidence is still required. Br J Anaesth.

[12] Punnoose A., Claydon-Mueller L.S., Weiss O., Zhang J., Rushton A., Khanduja V. (2023). Prehabilitation for patients undergoing orthopedic surgery: a systematic review and meta-analysis. JAMA Netw Open.

[13] Hanna T.P., King W.D., Thibodeau S. (2020). Mortality due to cancer treatment delay: systematic review and meta-analysis. BMJ.

[14] Molenaar C.J.L., Minnella E.M., Coca-Martinez M. (2023). Effect of multimodal prehabilitation on reducing postoperative complications and enhancing functional capacity following colorectal cancer surgery: the PREHAB randomized clinical trial. JAMA Surg.

[15] Skořepa P., Ford K.L., Alsuwaylihi A. (2024). The impact of prehabilitation on outcomes in frail and high-risk patients undergoing major abdominal surgery: a systematic review and meta-analysis. Clin Nutr.

[16] Hawkins T., Agarwal S., Evans C.R. (2023). Centre for perioperative care anaemia guideline: implications for anaesthesia. Br J Anaesth.

[17] Shander A., Corwin H.L., Meier J. (2023). Recommendations from the international consensus conference on anemia management in surgical patients (ICCAMS). Ann Surg.

[18] Richards T., Baikady R.R., Clevenger B. (2020). Preoperative intravenous iron to treat anaemia before major abdominal surgery (PREVENTT): a randomised, double-blind, controlled trial. Lancet.

[19] Talboom K., Borstlap W.A.A., Roodbeen S.X. (2023). Ferric carboxymaltose infusion versus oral iron supplementation for preoperative iron deficiency anaemia in patients with colorectal cancer (FIT): a multicentre, open-label, randomised, controlled trial. Lancet Haematol.

[20] Myles P.S., Richards T., Klein A. (2022). Postoperative anaemia and patient-centred outcomes after major abdominal surgery: a retrospective cohort study. Br J Anaesth.

[21] Kougias P., Sharath S., Mi Z., Biswas K., Mills J.L. (2019). Effect of postoperative permissive anemia and cardiovascular risk status on outcomes after major general and vascular surgery operative interventions. Ann Surg.

[22] Verdonk F., Einhaus J., Tsai A.S. (2021). Measuring the human immune response to surgery: multiomics for the prediction of postoperative outcomes. Curr Opin Crit Care.

[23] Rumer K.K., Hedou J., Tsai A. (2022). Integrated single-cell and plasma proteomic modeling to predict surgical site complications: a prospective cohort study. Ann Surg.

[24] Patel A.B.U., Weber V., Gourine A.V., Ackland G.L. (2022). The potential for autonomic neuromodulation to reduce perioperative complications and pain: a systematic review and meta-analysis. Br J Anaesth.

[25] de la Motte L., Kehlet H., Vogt K. (2014). Preoperative methylprednisolone enhances recovery after endovascular aortic repair: a randomized, double-blind, placebo-controlled clinical trial. Ann Surg.

[26] Bain C.R., Myles P.S., Corcoran T., Dieleman J.M. (2023). Postoperative systemic inflammatory dysregulation and corticosteroids: a narrative review. Anaesthesia.

[27] Nielsen N.I., Kehlet H., Gromov K. (2022). High-dose steroids in high pain responders undergoing total knee arthroplasty: a randomised double-blind trial. Br J Anaesth.

[28] Carley M.E., Chaparro L.E., Choiniere M. (2021). Pharmacotherapy for the prevention of chronic pain after surgery in adults: an updated systematic review and meta-analysis. Anesthesiology.

[29] Beloeil H., Garot M., Lebuffe G. (2021). Balanced opioid-free anesthesia with dexmedetomidine versus balanced anesthesia with remifentanil for major or intermediate noncardiac surgery. Anesthesiology.

[30] Chassery C., Atthar V., Marty P. (2024). Opioid-free versus opioid-sparing anaesthesia in ambulatory total hip arthroplasty: a randomised controlled trial. Br J Anaesth.

[31] Leger M., Perrault T., Pessiot-Royer S. (2024). Opioid-free anesthesia protocol on the early quality of recovery after major surgery (SOFA trial): a randomized clinical trial. Anesthesiology.

[32] Kharasch E.D., Clark J.D. (2021). Opioid-free anesthesia: time to regain our balance. Anesthesiology.

[33] Thiele R.H., Raghunathan K., Brudney C.S. (2016). American Society for Enhanced Recovery (ASER) and Perioperative Quality Initiative (POQI) joint consensus statement on perioperative fluid management within an enhanced recovery pathway for colorectal surgery. Perioper Med (Lond).

[34] Miller T.E., Myles P.S. (2019). Perioperative fluid therapy for major surgery. Anesthesiology.

[35] Lobo D.N. (2023). The 2023 Sir David Cuthbertson Lecture. A fluid journey: experiments that influenced clinical practice. Clin Nutr.

[36] Myles P.S., Bellomo R., Corcoran T. (2018). Restrictive versus liberal fluid therapy for major abdominal surgery. N Engl J Med.

[37] Pinsky M.R., Cecconi M., Chew M.S. (2022). Effective hemodynamic monitoring. Crit Care.

[38] Saugel B., Thomsen K.K., Maheshwari K. (2023). Goal-directed haemodynamic therapy: an imprecise umbrella term to avoid. Br J Anaesth.

[39] Jessen M.K., Vallentin M.F., Holmberg M.J. (2022). Goal-directed haemodynamic therapy during general anaesthesia for noncardiac surgery: a systematic review and meta-analysis. Br J Anaesth.

[40] Rollins K.E., Lobo D.N. (2016). Intraoperative goal-directed fluid therapy in elective major abdominal surgery: a meta-analysis of randomized controlled trials. Ann Surg.

[41] Rollins K.E., Mathias N.C., Lobo D.N. (2019). Meta-analysis of goal-directed fluid therapy using transoesophageal doppler monitoring in patients undergoing elective colorectal surgery. BJS Open.

[42] Scott M.J., APSF Hemodynamic Instability Writing Group (2023). Perioperative patients with hemodynamic instability: consensus recommendations of the anesthesia patient safety foundation. Anesth Analg.

[43] Aldecoa C., Bettelli G., Bilotta F. (2024). Update of the European society of anaesthesiology and intensive care medicine evidence-based and consensus-based guideline on postoperative delirium in adult patients. Eur J Anaesthesiol.

[44] Joris J., Kehlet H., Slim K. (2022). Postoperative cognitive dysfunction: time for enhanced recovery after surgery programmes. Eur J Anaesthesiol.

[45] Majeed A.W., Troy G., Nicholl J.P. (1996). Randomised, prospective, single-blind comparison of laparoscopic versus small-incision cholecystectomy. Lancet.

[46] Basse L., Jakobsen D.H., Bardram L. (2005). Functional recovery after open versus laparoscopic colonic resection: a randomized, blinded study. Ann Surg.

[47] Maibom S.L., Roder M.A., Aasvang E.K. (2022). Open vs robot-assisted radical cystectomy (BORARC): a double-blinded, randomised feasibility study. BJU Int.

[48] Joshi G.P., Stewart J., Kehlet H. (2022). Critical appraisal of randomised trials assessing regional analgesic interventions for knee arthroplasty: implications for postoperative pain guidelines development. Br J Anaesth.

[49] Joshi G.P., Albrecht E., Van de Velde M., Kehlet H., Lobo D.N. (2023). Prospect working group of the European society of regional anaesthesia pain therapy. PROSPECT methodology for developing procedure-specific pain management recommendations: an update. Anaesthesia.

[50] Levy N., Quinlan J., El-Boghdadly K. (2021). An international multidisciplinary consensus statement on the prevention of opioid-related harm in adult surgical patients. Anaesthesia.

[51] Baamer R.M., Iqbal A., Lobo D.N., Knaggs R.D., Levy N.A., Toh L.S. (2022). Utility of unidimensional and functional pain assessment tools in adult postoperative patients: a systematic review. Br J Anaesth.

[52] Huang L., Kehlet H., Petersen R.H. (2022). Reasons for staying in hospital after video-assisted thoracoscopic surgery lobectomy. BJS Open.

[53] Huang L., Kehlet H., Petersen R.H. (2022). Functional recovery after discharge in enhanced recovery video-assisted thoracoscopic lobectomy: a pilot prospective cohort study. Anaesthesia.

[54] Wainwright T.W., Kehlet H. (2022). Functional recovery following hip and knee arthroplasty - subjective vs objective assessment?. Acta Orthop.

[55] Huang L., Frandsen M.N., Kehlet H., Petersen R.H. (2022). Days alive and out of hospital after enhanced recovery video-assisted thoracoscopic surgery lobectomy. Eur J Cardio Thorac Surg.

[56] Jørgensen C.C., Kehlet H. (2017). Lundbeck foundation centre for fast-track hip, knee replacement collaborative group. Time course and reasons for 90-day mortality in fast-track hip and knee arthroplasty. Acta Anaesthesiol Scand.

[57] BBC News (2015). https://www.bbc.co.uk/news/magazine-34247629.

[58] Joshi G.P., Alexander J.C., Kehlet H. (2018). Large pragmatic randomised controlled trials in peri-operative decision making: are they really the gold standard?. Anaesthesia.

[59] Myles P.S., Yeung J., Beattie W.S., Ryan E.G., Heritier S., McArthur C.J. (2023). Platform trials for anaesthesia and perioperative medicine: a narrative review. Br J Anaesth.

[60] Varadhan R., Seeger J.D., Velentgas P., Dreyer N.A., Nourjah P. (2013). Developing a protocol for observational comparative effectiveness research: a user's guide.

[61] Giraudeau B., Caille A., Eldridge S.M., Weijer C., Zwarenstein M., Taljaard M. (2022). Heterogeneity in pragmatic randomised trials: sources and management. BMC Med.

