# Supplementary material for: Exploring the need for reconsideration of trial design in perioperative outcomes research: a narrative review
Source: eClinicalMedicine. 2024 Feb 29;70:102510. doi: 10.1016/j.eclinm.2024.102510 (PMC10912044; doi:10.1016/j.eclinm.2024.102510)
Supplement: Supplementary 1 [file mmc1.docx]

**Supplementary 1.**

**Some useful websites where readers can find further information on the subject**

Enhanced Recovery After Surgery (ERAS®) Society <https://erassociety.org>

American Society of Enhanced Recovery (ASER) <https://www.aserhq.org/web/>

PeriOperative Quality Initiative (POQI) <https://thepoqi.org>

PROcedure SPEcific postoperative pain managemenT (PROSPECT) <https://esraeurope.org/prospect/>

Centre for Perioperative Care (CPOC) <https://cpoc.org.uk>

Perioperative Medicine: The Pathway to Better Surgical Care <https://www.rcoa.ac.uk/sites/default/files/documents/2019-08/Perioperative%20Medicine%20-%20The%20Pathway%20to%20Better%20Care.pdf>

The Society for Perioperative Assessment and Quality Improvement (SPAQI) <https://www.spaqi.org/web/index.php>

UK Perioperative Medicine Clinical Trials Network <https://www.rcoa.ac.uk/research/research-bodies/perioperative-medicine-clinical-trials-network>

ANZCA Perioperative Medicine Special Interest <https://www.anzca.edu.au/fellowship/anaesthesia-continuing-education-(1)/perioperative-medicine-special-interest-group>

ANZCA Library: Perioperative Medicine <https://libguides.anzca.edu.au/perioperative>
